# Risk Factors for Surgical Site Infections Following Fasciotomy in Patients With Acute Compartment Syndrome: A Study on the February 2023 Kahramanmaraş Earthquake

**DOI:** 10.7759/cureus.46880

**Published:** 2023-10-11

**Authors:** Erkan Akgun, Abdulsamet Emet, Kemal Sibar, Faruk M Çatma, Ismail Aykut Kocyigit, Ali Şahin, Emrah Imat, Ibrahim Faruk Adiguzel, Ahmet Fırat

**Affiliations:** 1 Orthopaedics and Traumatology, Ankara Etlik City Hospital, Ankara, TUR

**Keywords:** creatinine kinase, infection, fasciotomy, acute compartment syndrome, earthquake

## Abstract

Introduction: Surgical site infections (SSIs) developing after fasciotomy are difficult to treat, costly, and an important source of mortality and morbidity. This study aimed to determine the risk factors affecting the development of SSI in patients who underwent fasciotomy with the diagnosis of acute compartment syndrome (ACS) within 72 hours after two consecutive earthquakes of 7.7 and 7.6 magnitude that occurred in Kahramanmaraş on February 6, 2023.

Method: A total of 116 patients were retrospectively analyzed. Patients were divided into two groups: those who developed SSI and those who did not. In this study, variables such as basic demographic characteristics, time of fasciotomy, center performing fasciotomy, type of wound closure, affected extremity, concomitant renal failure, hyperbaric oxygen (HBO) therapy, blood creatine kinase (CK) level were examined.

Results: Of 116 patients, 58 (50%) had SSI. It was statistically observed that patients who underwent treatment with vacuum-assisted closure (VAC), those who underwent primary closure with the shoelace method, those who went into renal failure, and those whose fasciotomy was performed in an earthquake zone had a higher incidence of SSI (p<0.001). Blood CK level above 17.839 seemed to be a risk factor according to receiver operating characteristic (ROC) analysis (P<0.01). Age (p=0.193), gender (p=0.125), fasciotomy time (p=0.843), lower extremity (p=0.234), upper extremity (p=0.806), and HBO treatment (p=0.56) were not associated with SSI. Infection was found to be a significant risk factor for amputation (p<0.001).

Conclusion: The use of VAC as a wound closure technique for SSI after fasciotomy in patients who developed ACS due to the earthquake, the presence of renal failure in the patients, and performing fasciotomy in the earthquake zone were independent risk factors. A blood CK level above 17.839 was also determined as a risk factor, but the confidence interval was found to be low.

## Introduction

An earthquake is an unpredictable natural event, even if its location can be predicted. When the number of people who died in natural disasters in the last two decades is evaluated, more than half of them died in earthquakes. The number of light, moderate, or severe injuries varies depending on the population density in any affected area. On February 6, 2023, two major earthquakes of magnitude 7.7 and 7.6, centered in Kahramanmaraş, Turkey affected 11 provinces. It is estimated that over 50,000 people died, and more than twice as many were injured. When earthquake injuries are evaluated, these injuries can range from simple soft tissue trauma to closed or open fractures, compartment or crush syndrome, multiple fractures involving all four extremities, spinal and pelvic fractures, and even severe life-threatening trauma. These injuries may require treatment ranging from simple orthoses to complex surgical treatments.

Acute compartment syndrome (ACS) is a devastating condition frequently encountered by trauma surgeons, especially during earthquakes. Pathophysiologically, ACS begins with impaired blood circulation due to increased interstitial pressure, followed by decreased venous drainage. A vicious cycle results in increased interstitial pressure, decreased arterial blood flow, and eventually tissue necrosis, irreversible nerve damage, and even ischemia, leading to the need for amputation [1,2]. Diagnosing ACS is difficult, and there is no clinical consensus. Previous studies have shown that the clinical picture and repeated intracompartmental pressure measurements can be used for diagnosis [3,4]. When previous studies are analyzed, we see that ACS presents with clinical presentations that we call the 5Ps (pallor, severe pain, paresthesia, pulselessness, and paralysis). However, not all of these findings occur at the same time. Especially, pale extremities and absence of pulse are findings that occur late and indicate necrosis and ischemia [5,6]. In the diagnosis of ACS in unconscious patients, it is necessary to benefit from paraclinical applications such as intracompartmental pressure measurement [7,8]. However, measurement of intracompartmental pressure is a difficult and person-dependent method. Although there is no exact threshold for ACS, the common opinion is that the diagnosis can be made if the intracompartmental pressure is less than 30 mmHg lower than the diastolic pressure [6-8].

Rapid diagnosis and early fasciotomy is the gold standard approach in ACS [9,10]. Even fasciotomies performed under favorable conditions have a high rate of complications, and therefore, post-fasciotomy care should be maintained very carefully. The most important complication of fasciotomy after ACS is surgical site infection (SSI). This rate varies between 5% and 30% in different studies [6,11,12]. However, reports in recent years indicate that this rate has increased up to 36% [4,13,14]. SSIs that develop after fasciotomy can lead to more serious conditions such as limb loss, sepsis, and even death.

This study aimed to determine the risk factors by examining various independent variables before and after fasciotomy, especially in devastating national disasters such as earthquakes, where even public health systems are challenged.

## Materials and methods

Data collection

The study was initiated following the approval of the local ethics committee (Etlik City Hospital Ethics Committee with decision no: 2023/399). On February 6, 2023, as a result of two consecutive 7.7 and 7.6 magnitude earthquakes centered in Kahramanmaraş (Turkey), the medical records of 116 patients who underwent fasciotomies for ACS at the health bases in the earthquake zone or directly in our hospital and whose treatment continued in our hospital afterward were retrospectively reviewed. The inclusion criteria were patients diagnosed with ACS of the upper or lower extremities, had undergone fasciotomy, had post-fasciotomy treatment and care continued in our hospital, and whose time between the earthquake and fasciotomy did not exceed 72 hours. Patients were divided into two groups. The first group included patients with SSI, and the second group included patients without SSI after fasciotomy. Inclusion criteria for the SSI group were a positive intraoperative culture at the surgical site during any debridement within 30 days after fasciotomy, pathology samples of microbiological pathogens, and reoperation due to infection [15,16]. In both groups of patients, variables thought to be related, such as age, gender, lower extremity, upper extremity, time of fasciotomy, center performing fasciotomy, type of wound closure, renal failure, hyperbaric oxygen therapy (HBO), blood creatine kinase (CK) level were retrospectively analyzed via digital data system.

Follow-up and treatment

**Figure 1:**
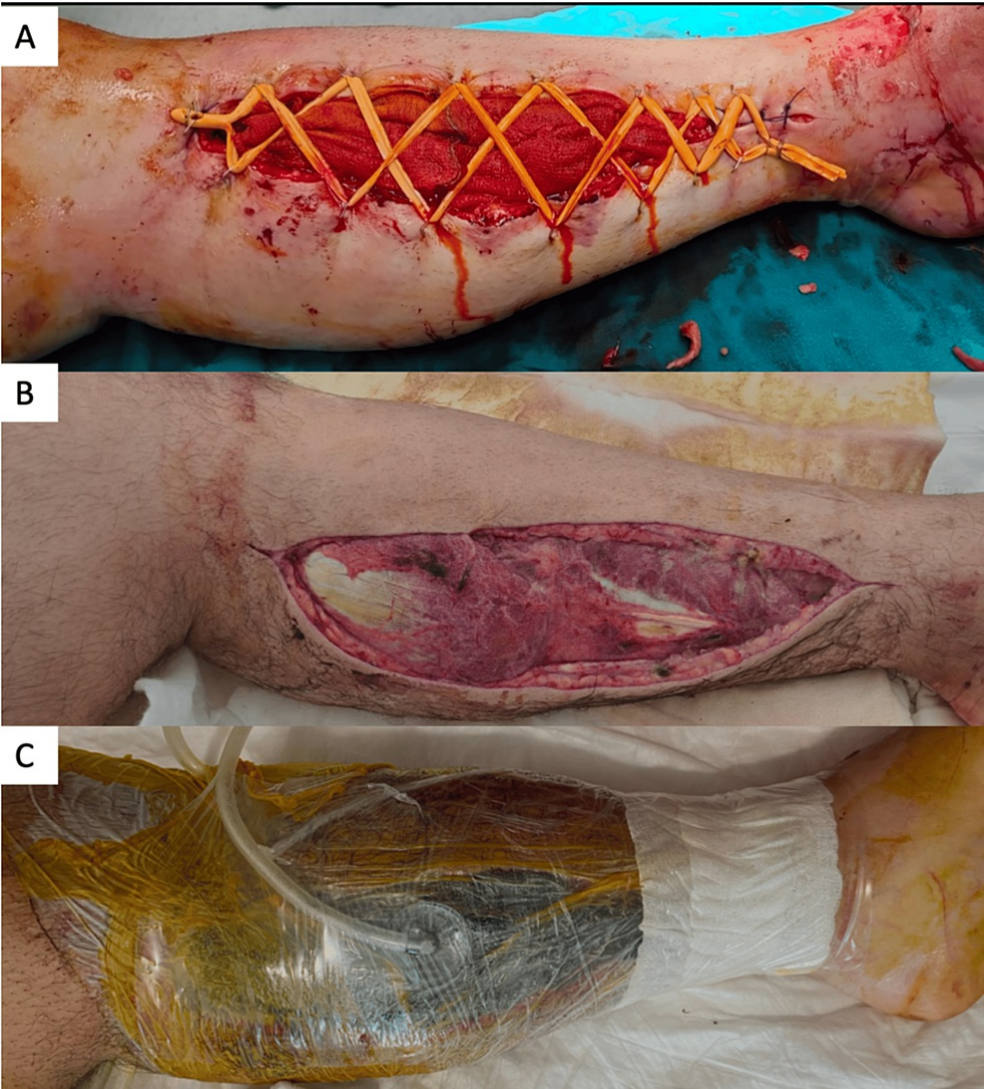
Clinical images of the patients. (A) Primary wound closure with shoelace method. (B) A patient with SSI whose fasciotomy was performed in on-site health camps in the earthquake zone. (C) Primary wound closure who underwent treatment with vacuum-assisted closure (VAC). SSI: surgical site infection

All patients were given intravenously first-generation cephalosporin prophylactically after admission, during, and for three days after the procedure. After fasciotomy, two different wound closure methods, primary closure with the shoelace method (PCSM) and vacuum-assisted closure (VAC), were used (Figure 1). In 59 patients who underwent PCSM, washing and debridement were performed in the operating room every two days after fasciotomy. The patients' fasciotomy incisions began to be closed by stretching, and primary closure was achieved in 39 patients (66%) in an average of 12±4.1 days. In 57 patients who underwent the VAC method, 100mm-Hg pressure was applied in continuous mode immediately after fasciotomy, and the wound was closed with VAC again after debridement in the operating room every three days. Primary closure was achieved in 12 patients with VAC in an average of 26±6.1 days.

Statistical analysis

Statistical Package for the Social Sciences (IBM SPSS Statistics for Windows, IBM Corp., Version 25.0, Armonk, NY) was used for statistical analysis. Descriptive statistics are presented as numbers and percentages. The Kolmogorov-Smirnow test was used for normality analysis. Measurement data were expressed as mean, standard deviation or median, and interquartile range (IQR: 25th-75th percentile) according to the parametric distribution of the variables. The chi-square test was used to compare categorical variables between groups. Receiver operating characteristic (ROC) analysis was applied to determine the cut-off point between CK and the SSI risk and the sensitivity and specificity at this cut-off point. Binary logistic regression analysis was performed to determine the variables associated with the risk of SSI. For statistical significance, p<0.05 was accepted, and data are presented as odds ratio (OR) and 95% confidence interval (CI).

## Results

This study included 116 patients (70 males, 46 females; mean age: 27.5 ± 16 years; range: 2-63 years) who were diagnosed with ACS with or without additional trauma and underwent fasciotomy after two consecutive 7.7 and 7.6 large earthquakes centered in Kahramanmaraş in February 2023. SSI requiring additional medical care and surgical intervention after fasciotomy developed in 58 patients (50%) (Table 1). The remaining 58 patients (50%) did not experience any infection-related complications.

**Table 1:** Total number of patients presentation of the diagnosis regarding injuries SSI: surgical site infection

Number of Patients	Isolated Crush Injury and Acute Compartment Syndrome	Isolated Additional Trauma	Multitrauma Patients
Total (116)	83	21	12
Patients with SSI (58)	42	9	7
		Humerus Fracture (1) Radius and Ulna Fracture (1) Femur Fracture (2) Tibia Fracture (4) Foot Injury (1)	Vertebral Fracture+Acetabulum+Pelvic Ring+Hemothorax+Upper or/and Lower Extremity Fractures
Patients without SSI (58)	41	12	5
		Humerus Fracture (2) Radius and Ulna Fracture (2) Femur Fracture (3) Tibia Fracture (2) Foot Injury (2)	Vertebral Fracture+Acetabulum+Pelvic Ring+Hemothorax+Upper or/and Lower Extremity Fractures

Demographic and clinical characteristics of the groups with and without SSI after fasciotomy and statistical comparisons between the groups are presented in Table 2. Accordingly, SSI was statistically higher in patients whose wound closure technique was VAC compared to those whose wound closure technique was PCSM (p<0.001), in patients who developed acute renal failure compared to those who did not (p< 0.001), and in those whose fasciotomy was performed in on-site health camps in the earthquake zone compared to those whose fasciotomy was performed in our hospital. In addition, increased blood CK level was found to be a variable associated with increased SSI (p<0.01). There was no statistically significant difference in infection rates between the two groups in independent variables such as age, gender, lower extremity, upper extremity, time of fasciotomy, and HBO therapy.

**Table 2:** Demographic and total data of earthquake patients IQR: interquartile range, SSI: surgical site infection, HBO: hyperbaric oxygen treatment, CK: creatine kinase, VAC: vacuum-assisted closure

	SSI Group (n=58)	Non-SSI Group (n=58)	P
Age (mean ± SD)	30.4±16.3	24.6±15.3	0.193
Gender (n/%)			0.744
Female	24 (41.4)	22 (37.9)	
Male	34 (58.6)	36 (62.1)	
Fasciotomy time (Hour) (median IQR)	25.5 (33.0)	24.0 (35.0)	0.680
Fasciotomy Hospital			<0.001
Etlik City Hospital	18 (30,5)	41 (69.5)	
Outer Center	40 (70.2)	17 (29.8)	
Upper Extremity SSI	19 (32.8)	-	0.806
Unilateral	15 (78.9)	-	
Bilateral	4 (21.1)	-	
Lower Extremity SSI	49 (84.5)	-	0.239
Unilateral	36 (62.1)	-	
Bilateral	13 (37.9)	-	
Amputation			<0.001
+	26 (44.8)	8 (13.8)	
-	32 (55.2)	50 (86.2)	
Surgical Technique			<0.001
Shoelace	16 (27.6)	43 (74.1)	
VAC	42 (72.4)	15 (25.9)	
Renal Failure during Follow-up			<0.001
Renal Failure +	36 (62.1)	18 (31.0)	
Renal Failure -	22 (37.9)	40 (69.0)	
HBO			0.560
HBO treatment +	19 (32.8)	22 (37.9)	
HBO treatment -	39 (67.2)	36 (62.1)	
CK	23868.50 (72588)	11812.50 (54877)	<0.01

When the wound closure methods were compared between the group who developed SSI and those who did not, it was observed that SSI developed statistical significance in patients who underwent the VAC method (p<0.001). Binary logistic regression analysis also showed a strong association between the risk of SSI and the VAC method (p<0.01, OR: 7.561, 95% CI (1.546-11.235)). The other independent variable, the presence of renal failure, was statistically significantly associated with SSI (p<0.001). In binary logistic regression analysis, renal failure significantly increased the likelihood of SSI (p<0.01, OR: 5.721, 95% CI (0.701-46.681)). Another significant difference between the groups was the center of fasciotomy. Patients who underwent fasciotomy at hospitals in the earthquake zone developed higher SSI (p<0.001). Regression analysis also showed that fasciotomies performed in the earthquake zone posed a higher risk of SSI compared to fasciotomies performed in our hospital (p<0.01, OR: 5.343, 95% CI (1.243-9.449)).

**Figure 2:**
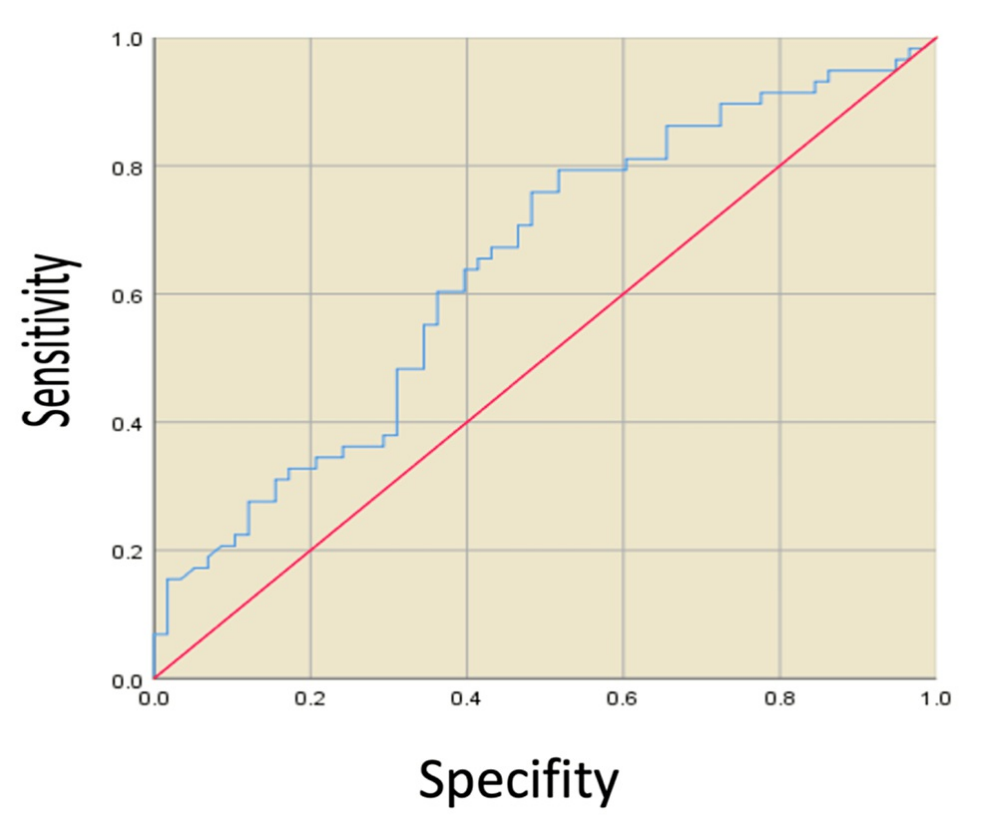
ROC curve for CK and risk of surgical site infection ROC: receiver operating characteristic, CK: creatine kinase

ROC analysis was performed for blood CK level, and it was found that it could be an independent variable for SSI above the cut-off value of 17.839, as shown in Figure 2, Table 3 (p<0.001). However, both the sensitivity and specificity of this analysis were found to be 60.3%. Age (p=0.193), gender (p=0.744), time of fasciotomy (p=0.680), upper extremity (p=0.806), lower extremity (p=0.239), and HBO therapy (p=0.560) were not statistically associated with SSI and were not found to be risk predictors in logistic regression analysis.

**Table 3:** ROC analysis for creatine kinase and surgical site infection risk ROC: receiver operating characteristic, CK: creatine kinase, VAC: vacuum-assisted closure

Risk Factor	VAC (95% CI)	Cut-off	P	Sensitivity (%)	Specificity (%)
CK	.543-.794	17.839	<0.01	60.3	60.3

In our study, amputation was required in 26 of 58 patients (44.8%) in the group with SSI despite serial debridement and antibiotic treatment, while this rate was limited to eight patients (13.8%) in the group without SSI. It was statistically shown that amputation was performed more frequently in the group with SSI (p<0.001).

## Discussion

In our study, 58 (50%) of 116 patients who underwent fasciotomy for ACS developed SSI. This rate seems to be high when compared with previous studies. In the literature, the rate of SSI after fasciotomy is up to 30% [17-19]. However, most of these studies are data from patients who underwent fasciotomy for ACS independent of the earthquake. The number of studies investigating SSI rates in patients who underwent earthquake-related fasciotomy is quite low because we believe that the presence of many independent variables, unintended ACS in healthcare systems, deficiencies, and inadequacies in patient records make it difficult to determine infection rates in such national disaster periods. This is the first study focusing on investigating the independent variables that may be risk determinants for the development of SSI in patients who were diagnosed with ACS due to earthquake and underwent fasciotomy.

Although many countries create emergency action plans for such natural disasters, the timing and severity of earthquakes cannot be predicted in advance, which creates serious stress and excessive workload on the health systems of countries. It is known that fasciotomy performed following the technique and in the early period of the treatment of ACS is both limb and life-saving, but treatment is not limited to fasciotomy; wound care in the post-fasciotomy period is also just as important [17,20,21]. SSI developed during this period significantly increases patient mortality and morbidity rates [11,15,18]. Postoperative infections result in sepsis, amputation, and even death in patients. Therefore, we believe it is important to investigate the risk determinants of SSI and take preventive measures due to its high prevalence and poor prognosis.

Considering the demographic characteristics of the patients, it was observed that age was not an effective factor in the development of SSI. Generally, the same results were obtained in previous studies [4,20]. Different views are provided regarding another characteristic, gender. Some studies have shown higher rates of SSI in the male gender, while others have not found a relationship [4,19,20,22]. In this study, no difference was found between male and female gender in terms of SSI. Likewise, no significant correlation was found between the two groups when the time of fasciotomy and the extremity of fasciotomy (upper extremity versus lower extremity) were analyzed. Merchan et al. obtained similar results in a retrospective study of 142 patients and did not find a relationship between the time of fasciotomy and the site of injury and SSI [20]. This study found that fasciotomies performed for ACS treatment within 72 hours of the earthquake did not increase SSI infection and were not a risk predictor.

Another independent variable, adjuvant HBO treatment in the postfasiotomy period, has positively affected the healing and survival of the tissues left behind after fasciotomy [23,24]. It is also argued that it has a toxic effect on microbiological agents by providing high oxygen concentration in tissues [25]. In this study, 19 patients (32.8%) in the group who developed SSI and 22 patients (39.7%) in the group who did not develop SSI received postfasiotomy adjuvant HBO therapy. However, the relationship between HBO treatment and the development of SSI was not found between the two groups.

Our study found that one of the independent variables associated with SSI after fasciotomy was those performed in local or field hospitals in the earthquake zone. When we compared the fasciotomies performed in health institutions in the earthquake zone with the fasciotomies performed in our hospital, we observed a 5.343-fold increased risk of SSI. We could not find a study on this variable in the literature. The 2023 Kahramanmaraş earthquake caused serious damage in an area of 350 thousand square meters in addition to its severity and long duration. As a result, serious damage occurred in health centers and transportation to these centers in Kahramanmaraş and many neighboring provinces, and it was also shown that many personnel working in these health centers were also affected by the earthquake. Although the Republic of Turkey quickly established field hospitals on land and sea and dispatched health personnel to the region to fill this gap, the conditions of the region, the inability to provide appropriate operating rooms and sterility conditions, and patient urgency may have significantly increased the SSI rate in fasciotomies performed in this region. On the other hand, Ankara Etlik City Hospital, with its 3,625 bed capacity, 125 operating rooms, and sufficient healthcare personnel, provided intervention to patients in more appropriate operating rooms and sterility conditions away from the deficiencies of the earthquake zone and, therefore, less SSI may have been encountered in fasciotomies performed here.

Another risk determinant was using the VAC technique as a wound closure technique. Two different wound closure techniques were applied to the patients in the postfasiotomy process. Patients who underwent VAC wound closure had a 7.56 times higher risk of SSI compared to patients who underwent PCSM. In addition, the wound closure time was longer with the VAC technique, and more patients required grafts requiring additional surgery. There is no consensus in the literature on the best method for wound closure after fasciotomy [26-28]. A meta-analysis study by Jauregui et al. demonstrated that the VAC technique had the lowest complication rate in wound closure after fasciotomy, but the success rate was lower than the PCSM technique [27]. Another study claimed that closure with the VAC technique prolonged the time required for wound closure and increased the complications related to it [29]. The study by Hake et al. found that closure with the VAC technique was an important risk determinant in the development of SSI [19]. In this study, primary complete closure was achieved in 39 (66%) patients who underwent PCSM in an average of 12±4.1 days, and SSI developed in only 16 (27.6%) of the patients. However, complete primary closure was achieved in only 12 (21%) patients who underwent VAC in a mean of 26±6.1 days, and SSI developed in 42 patients (72.4%) in this group.

Another determinant variable in the development of SSI was blood CK level. Blood CK level was significantly higher in the SSI group compared to the non-SSI group. Previous studies have shown that blood CK level may be a marker in diagnosing ACS and evaluating muscle necrosis [30,31]. However, no study shows an association between blood CK levels and SSI. When we analyzed the data in ROC curve analysis, we calculated the cut-off value of blood CK level as 17.839. The presence of renal failure was also found to be associated with SSI after fasciotomy. Based on our study, patients with renal insufficiency after fasciotomy had a 5.721 times higher risk of SSI compared to patients without renal insufficiency, which was similar to the literature. The general susceptibility to infection in patients with renal failure has been previously shown in many studies [32]. Our study also supports this.

Although not the primary outcome measure, another significant result in this study was the high amputation rate in the group with SSI. Amputation was performed in 26 patients (44.8%) in the group with SSI compared to only eight patients (13.8%) in the group without SSI. Amputation rates in patients who develop SSI after fasciotomy are reported to be as high as 35% [10,20]. According to the results of the study, the development of SSI after fasciotomy was shown to increase the possible risk of amputation by 4.936.

Although this is the first study investigating risk factors for SSI in patients who underwent fasciotomy after earthquake-related ACS, it has some limitations. First, this is a retrospective study conducted in a single institution. In addition, our patients could not be homogenized sufficiently as people from all populations were affected by the earthquake. Another limitation is that although patients were clinically diagnosed with ACS, compartment measurement, which is an objective diagnostic criterion, could not be performed in every patient. Finally, the sample size of this study was relatively small. A clinical study with a larger sample size was necessary.

## Conclusions

In conclusion, in our study, closure with the VAC technique, fasciotomies performed in health centers in the earthquake zone, and the presence of renal failure were independent risk factors of SSI. The blood CK above 17.839 predicted the risk of SSI, but its specificity and sensitivity were 60.3%.

## References

[1] Köstler W, Strohm PC, Südkamp NP (2005). Acute compartment syndrome of the limb. Injury.

[2] Janzing HM (2007). Epidemiology, etiology, pathophysiology and diagnosis of the acute compartment syndrome of the extremity. Eur J Trauma Emerg Surg.

[3] O'Toole RV, Whitney A, Merchant N, Hui E, Higgins J, Kim TT, Sagebien C (2009). Variation in diagnosis of compartment syndrome by surgeons treating tibial shaft fractures. J Trauma.

[4] Yang S, Long Y, Wang T, Guo J, Hou Z (2023). Predictors for surgical site infection after fasciotomy in patients with acute leg compartment syndrome. J Orthop Surg Res.

[5] Prayson MJ, Chen JL, Hampers D, Vogt M, Fenwick J, Meredick R (2006). Baseline comparment pressure measurements in isolated lower extremity fractures without clinical compartment syndrome. J Trauma.

[6] Hope MJ, McQueen MM (2004). Acute compartment syndrome in the absence of fracture. J Orthop Trauma.

[7] Shields RW Jr, Root KE Jr, Wilbourn AJ (1986). Compartment syndromes and compression neuropathies in coma. Neurology.

[8] Nudel I, Dorfmann L, deBotton G (2017). The compartment syndrome: is the intra-compartment pressure a reliable indicator for early diagnosis?. Math Med Biol.

[9] Rorabeck CH (1984). The treatment of compartment syndromes of the leg. J Bone Joint Surg Br.

[10] Rothenberg KA, George EL, Trickey AW, Chandra V, Stern JR (2019). Delayed fasciotomy is associated with higher risk of major amputation in patients with acute limb ischemia. Ann Vasc Surg.

[11] Rush DS, Frame SB, Bell RM, Berg EE, Kerstein MD, Haynes JL (1989). Does open fasciotomy contribute to morbidity and mortality after acute lower extremity ischemia and revascularization?. J Vasc Surg.

[12] Nehler MR, Hiatt WR, Taylor LM Jr (2003). Is revascularization and limb salvage always the best treatment for critical limb ischemia?. J Vasc Surg.

[13] Gourgiotis S, Villias C, Germanos S, Foukas A, Ridolfini MP (2007). Acute limb compartment syndrome: a review. J Surg Educ.

[14] Heemskerk J, Kitslaar P (2003). Acute compartment syndrome of the lower leg: retrospective study on prevalence, technique, and outcome of fasciotomies. World J Surg.

[15] Holihan JL, Flores-Gonzalez JR, Mo J, Ko TC, Kao LS, Liang MK (2017). How long is long enough to identify a surgical site infection?. Surg Infect (Larchmt).

[16] Mangram AJ, Horan TC, Pearson ML, Silver LC, Jarvis WR (1999). Guideline for prevention of surgical site infection, 1999. Centers for Disease Control and Prevention (CDC) Hospital Infection Control Practices Advisory Committee. Am J Infect Control.

[17] Velmahos GC, Theodorou D, Demetriades D (1997). Complications and nonclosure rates of fasciotomy for trauma and related risk factors. World J Surg.

[18] Oncül O, Keskin O, Acar HV (2002). Hospital-acquired infections following the 1999 Marmara earthquake. J Hosp Infect.

[19] Hake ME, Etscheidt J, Chadayammuri VP, Kirsch JM, Mauffrey C (2017). Age and dressing type as independent predictors of post-operative infection in patients with acute compartment syndrome of the lower leg. Int Orthop.

[20] Merchan N, Ingalls B, Garcia J, Wixted J, Rozental TD, Harper CM, Dowlatshahi AS (2022). Factors associated with surgical site infections after fasciotomy in patients with compartment syndrome. J Am Acad Orthop Surg Glob Res Rev.

[21] Ebraheim NA, Abdelgawad AA, Ebraheim MA, Alla SR (2012). Bedside fasciotomy under local anesthesia for acute compartment syndrome: a feasible and reliable procedure in selected cases. J Orthop Traumatol.

[22] Morris BJ, Unger RZ, Archer KR, Mathis SL, Perdue AM, Obremskey WT (2013). Risk factors of infection after ORIF of bicondylar tibial plateau fractures. J Orthop Trauma.

[23] Kirby JP (2019). Hyperbaric oxygen therapy emergencies. Mo Med.

[24] Weiland DE (2007). Fasciotomy closure using simultaneous vacuum-assisted closure and hyperbaric oxygen. Am Surg.

[25] Delaney JS, Montgomery DL (2001). How can hyperbaric oxygen contribute to treatment?. Phys Sportsmed.

[26] Ojike NI, Roberts CS, Giannoudis PV (2010). Compartment syndrome of the thigh: a systematic review. Injury.

[27] Jauregui JJ, Yarmis SJ, Tsai J, Onuoha KO, Illical E, Paulino CB (2017). Fasciotomy closure techniques. J Orthop Surg (Hong Kong).

[28] Kakagia D, Karadimas EJ, Drosos G, Ververidis A, Trypsiannis G, Verettas D (2014). Wound closure of leg fasciotomy: comparison of vacuum-assisted closure versus shoelace technique. A randomised study. Injury.

[29] Ihedioha U, Sinha S, Campbell AC (2005). Do creatine kinase (CK) levels influence the diagnosis or outcome in patients with compartment syndrome?. Scott Med J.

[30] Nilsson A, Alkner B, Wetterlöv P, Wetterstad S, Palm L, Schilcher J (2019). Low compartment pressure and myoglobin levels in tibial fractures with suspected acute compartment syndrome. BMC Musculoskelet Disord.

[31] Zhang L, Fu P, Wang L (2012). The clinical features and outcome of crush patients with acute kidney injury after the Wenchuan earthquake: differences between elderly and younger adults. Injury.

[32] Kazancioglu R, Cagatay A, Calangu S (2002). The characteristics of infections in crush syndrome. Clin Microbiol Infect.

